# Cultural Competence and Ethnic Diversity in Healthcare

**DOI:** 10.1097/GOX.0000000000002219

**Published:** 2019-05-16

**Authors:** Lakshmi Nair, Oluwaseun A. Adetayo

**Affiliations:** From the * Albany Medical College, Albany, N.Y.; † Division of Plastic Surgery, Albany Medical Center, Albany, N.Y.

## Abstract

Today’s model of healthcare has persistent challenges with cultural competency, and racial, gender, and ethnic disparities. Health is determined by many factors outside the traditional healthcare setting. These social determinants of health (SDH) include, but are not limited to, education, housing quality, and access to healthy foods. It has been proposed that racial and ethnic minorities have unfavorable SDH that contributes to their lack of access to healthcare. Additionally, African American, Hispanic, and Asian women have been shown to be less likely to proceed with breast reconstructive surgery post-mastectomy compared to Caucasian women. At the healthcare level, there is underrepresentation of cultural, gender, and ethnic diversity during training and in leadership. To serve the needs of a diverse population, it is imperative that the healthcare system take measures to improve cultural competence, as well as racial and ethnic diversity. Cultural competence is the ability to collaborate effectively with individuals from different cultures; and such competence improves health care experiences and outcomes. Measures to improve cultural competence and ethnic diversity will help alleviate healthcare disparities and improve health care outcomes in these patient populations. Efforts must begin early in the pipeline to attract qualified minorities and women to the field. The authors are not advocating for diversity for its own sake at the cost of merit or qualification, but rather, these efforts must evolve not only to attract, but also to retain and promote highly motivated and skilled women and minorities. At the trainee level, measures to educate residents and students through national conferences and their own institutions will help promote culturally appropriate health education to improve cultural competency. Various opportunities exist to improve cultural competency and healthcare diversity at the medical student, resident, attending, management, and leadership levels. In this article, the authors explore and discuss various measures to improve cultural competency as well as ethnic, racial, and gender diversity in healthcare.

By 2050, it is estimated that 50% of the US population will consist of minorities and unfortunately, today’s model of healthcare has been noted to have persistent racial and ethnic discrepancies.^1^ Diverse populations require personalized approaches to meet their healthcare needs. Minorities have been shown to have decreased access to preventive care and treatment for chronic conditions which results in increased emergency room visits, graver health outcomes, and increased likelihood of developing cardiovascular disease, diabetes, cancer, and mental illness.^2–5^

This disparity has been prominent in the field of plastic and reconstructive surgery. For example, Sharma et al. explains that there are significant racial disparities in breast reconstruction surgery. Specifically, African American, Hispanic, and Asian women are less likely to proceed with breast reconstructive surgery postmastectomy compared with White women. A study using the Surveillance, Epidemiology, and End Results database found that more African American women compared with White counterparts opted not to have immediate breast reconstruction after mastectomy, many stating they were unable to afford surgery. This discrepancy has been supported by future studies after Medicaid expansion and coverage.^1^

Health is determined by many factors outside the traditional healthcare setting. These social determinants of health (SDH) include housing quality, access to healthy food, and education.^6^ It has been proposed that racial and ethnic minorities have unfavorable SDH that contributes to their lack of access to healthcare.^6^ Differences in healthcare treatment and outcomes among minorities persist even after adjusting for socioeconomic factors.^3^ We hypothesize that lack of female and minority representation in the field of plastic surgery contributes to delayed healthcare and quality of outcomes in these populations. To be able to cater to these healthcare needs down the pipeline, it is critical that we begin efforts for attraction and retention of skilled female surgeons and minorities farther up in the pipeline chain. Although women compose half of all medical school graduates, only 14% of plastic surgeons and 32% of plastic surgery residents are women.^7^

The senior author (O.A.A.) wrote a response to Drs. Butler, Britt, and Longaker regarding the scarcity of ethnic diversity in plastic surgery in 2010. At that time, as a Black female in plastic and reconstructive surgery, O.A.A. represented a mere 3.7% of plastic and reconstructive surgery residents and fellows.^8^ It is astonishing that nearly a decade later we still face nearly identical statistics. It is imperative to prioritize diversity in plastic surgery so that by the next decade, we can make significant strides in narrowing this enormous disparity in representation. The authors are not advocating for diversity for its own sake at the cost of merit or qualification, but rather, that organizations and specialties initiate efforts to attract, retain, and promote highly motivated and skilled women and minorities.

Advocating for women and minorities in plastic surgery is one step in acknowledging and catering to various cultural differences. Culture is defined as a cumulative deposit of knowledge acquired by a group of people over the course of generations.^4^ Cultural competence is the ability to collaborate effectually with individuals from different cultures, and such competence can help improve healthcare experience and outcomes.^3,4^

Studies have identified limited national efforts to incorporate cultural competency in healthcare.^9^ In a national study of organizational efforts to reduce physician racial and ethnic disparities, 53% of organizations surveyed had 0–1 activities to reduce disparities out of over 20 possible actions to reduce disparities. Some examples of these disparity-reducing activities include providing educational materials in a different language, providing online resources to educate physicians on cultural competence, and awards at national meetings to recognize efforts to reduce racial disparities. The membership size of the national physician organization surveyed and the presence of a health disparities committee were found to be positively associated with organizations with at least 1 disparity-reducing activity. Primary care organizations were more likely to participate in disparity-reducing activities and may serve as role models for other physician organizations to take initiative.^9^

Various opportunities exist to improve cultural competency. One of such measures is via education of residents and students before they transition into attending roles. The Accreditation Council for Graduate Medical Education has identified the importance of addressing cultural diversity as part of its professionalism competency, and the Alliance of Continuing Medical Education also devoted lectures at its national annual conference to cultural competency.^10^ Such measures will help increase awareness in trainees and bridge the gap of competency as they transition from training to practice. Incorporating diversity training and cultural competence exercises at national plastic surgery meetings such as Plastic Surgery: The Meeting and AAPS with CME accreditation is a feasible way to incorporate this training. Additional efforts at the state and national level are also critical for advancing cultural competency, and some of these efforts are also underway.^6,10^ For instance, the Health and Human Services Office of Minority Health developed “Think Cultural Health,” a resource center that offers users the ability to earn continuing education credits in cultural competency through online case-scenario-based training.^6^ In addition, 5 states established legislature requiring or strongly recommending cultural competency training for physicians.^10^ These implementation efforts will help in raising awareness to improve cultural competency and diversity in healthcare.

On the industry level, the lack of diversity in healthcare leadership is dramatic, with 98% of senior management in healthcare organizations being White.^4^ This disparity in representation is similarly magnified when looking at minority representation in leadership roles within plastic surgery. Only 7% of department chiefs and chairs of plastic surgery are women. Improving representation of women and ethnic minorities in White-male dominated fields like plastic surgery has the potential to improve access to healthcare in minority populations. In fact, female leadership has even been associated with increased effectiveness.^11^

Even when individuals from racially or ethnically under-represented populations attain high level executive positions, most earn lower salaries and are overrepresented in management positions serving indigent populations.^12^ It is critical to address these gaps and disparities in healthcare. Some measures are being taken to attain culture competency via targeting upper-level executives to identify cultural competency as a high priority.^4,12^ Others propose targeting cultural competence in healthcare at the root, namely medical education. Some of the problematic themes identified include lack of exposure and insufficient education and teaching curricula regarding diversity; unfortunately, cultural competence is often perceived as a low priority in an overloaded academic curriculum.^13^

In the healthcare industry, efforts have been made to achieve cultural competence with the goal of providing culturally congruent care.^4^ A review of culturally competent healthcare industry systems identified 5 interventions to improve cultural competence: (1) gear programs to recruit and retain diverse staff members, (2) cultural competency training for healthcare providers, (3) use of interpreter services to ensure individuals from different backgrounds can effectively communicate, (4) culturally appropriate health education materials to inform staff of different cultural backgrounds, and (5) provision of culturally specific healthcare settings.^14^ Through increased awareness and by incorporating these interventions, culture competence can be improved in plastic surgery from bedside to the operating room.

Regrettably, there is a lack of literature linking culturally competent education to patient, professional, organizational outcomes. Horvat et al. created a 4-dimensional conceptual framework to assess intervention efficacy: educational content, pedagogical approach, structure of the intervention, and participant characteristics. It is essential that future studies follow methodologic rigor and reproducibility to best document progress.^15^

An examination of 119 California hospitals revealed that nonprofit hospitals serve more diverse patient populations, are in more affluent and competitive markets, and exhibit higher cultural competency. It is argued that there will be a market incentive for implementing culturally competent programs as long as cultural competency is linked to better patient experiences.^16^ Policymakers and institutions can capitalize on this and incorporate cultural competence practices into metrics for incentive payments. Additionally, enhancing public reporting on patient care and hospital quality will drive competition in the healthcare field and prompt organizations to aim for cultural competence.^16^

Striving for ethnic diversity and cultural competency in plastic surgery is necessary to adequately care for an evolving and diverse patient population. It is imperative that plastic surgery departments adopt evidence-based practices to foster cultural competency including promoting recruitment of diverse healthcare-providers, the use of interpreter services, cultural competency training for healthcare team members, and distribution of information on cultural competency to hospital staff members. As population demographics change, plastic surgery departments must also evolve to suit the needs of a diverse array of modern patients.
